# Carbon-Fiber-Reinforced PEEK Intramedullary Nails Defining the Niche

**DOI:** 10.1155/2019/1538158

**Published:** 2019-07-30

**Authors:** Georges F. Vles, Maximillian H. Brodermann, Mark A. Roussot, James Youngman

**Affiliations:** ^1^Department of Trauma and Orthopaedics, University College Hospital London, 235 Euston Rd, Fitzrovia, London NW1 2BU, UK; ^2^Department of Orthopaedic Surgery, University of Cape Town, Groote Schuur Hospital, Main Road, Observatory, Cape Town 7925, South Africa

## Abstract

**Background:**

Carbon-fiber-reinforced Polyetheretherketone (CFR-PEEK) nails are gaining interest as they have biomechanical properties potentially capable of overcoming disadvantages of conventional metal nails.

**Case Summary:**

Three cases are illustrated which required superior mechanical toughness, compatibility with radiotherapy, and postoperative advanced imaging.

**Conclusion:**

CFR-PEEK nails seem to have a niche role in distinct groups of patients.

## 1. Introduction

There is no doubt that metals like stainless steel, cobalt-chrome, and titanium alloys have changed the face of orthopaedics over the last decades. Nevertheless, technological progress in the field of biomaterials has led to the development of several high-potential composites that consist of a reinforcement material embedded within a matrix. One of the composites gaining particular interest is carbon-fiber-reinforced Polyetheretherketone (CFR-PEEK) [1] which has shown to be chemically inert and provoke minimal cellular response [2, 3]. Although evidence is still largely restricted to laboratory data, CFR-PEEK has shown to possess biomechanical properties potentially capable of overcoming disadvantages of traditional metal nails.

Firstly, controlled alignment of the carbon fibers can produce varying anisotropic properties so that the implant can be tailored to match the required biomechanical environment [1]. Therefore, its modulus of elasticity (3.5 GPa) better matches that of bone (1-20 GPa) compared to, for example, titanium nails (106 GPa) [4]. This theoretically leads to better callus formation and less stress shielding over time [5].

Secondly, fatigue failure can be a concern with conventional nails. This is the phenomenon of the nail and screws breaking if the bone does not heal in a timely fashion. Notorious examples are renal cell carcinoma metastatic fractures [6] or bisphosphonate-induced subtrochanteric femur fractures [7]. CFR-PEEK tibial nails have shown to withstand one million loading cycles (2240 N at a frequency of 5 Hz) without any visual signs of failure [8].

Thirdly, CFR-PEEK nails are radiolucent and sufficiently minimize artefacts on CT and MRI to allow assessment of immediate periprosthetic tissues [9]. This allows for better evaluation of fracture reduction and healing, detection of local recurrence or progression of pathological lesions, or subclinical infection. Furthermore, because of better planning and less interface effects due to increased homogeneity of the field, a lower irradiation dose may be required to reach the therapeutic threshold of adjuvant radiotherapy [10–12].

Finally, CFR-PEEK can offer a solution in patients with known metal allergies.

Although conventional metal nails will remain the gold standard for most long bone fixations, distinct groups of patients would benefit from the unique qualities associated with CFR-PEEK nailing, i.e., superior mechanical toughness and compatibility with radiotherapy and postoperative advanced imaging. We provide 3 case examples (CarboFix Orthopedics Ltd., Herzeliya, Israel) that illustrate the biomechanical properties and clinical applications of CFR-PEEK nails.

## 2. Case Presentation I: Superior Mechanical Toughness

**Figure 1:**
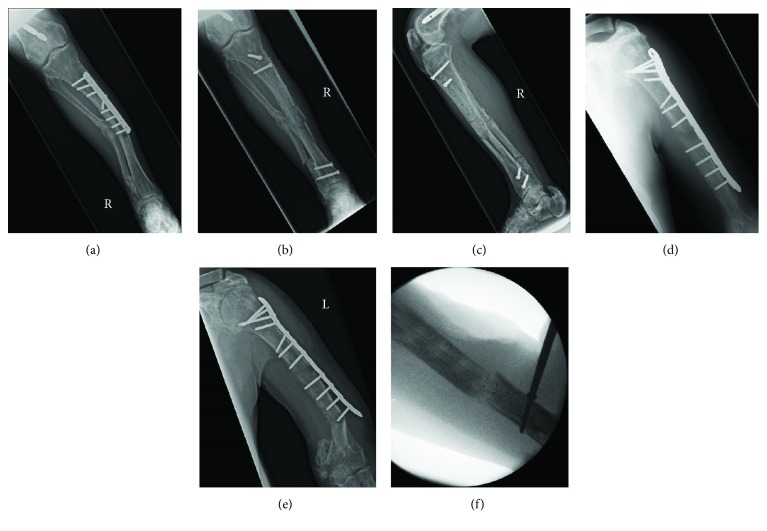
(a) Preoperative AP lower leg X-ray showing a periprosthetic fracture at the distal aspect of the metal plate with valgus deformity. (b, c) AP and lateral lower leg X-rays showing the stabilisation and restoration of alignment of the long bone with a tibial CFR-PEEK nail using proximal and distal locking screws. (d, e) Preoperative AP humerus X-rays showing failure of the metal plate and adjacent bone over time. (f) Intraoperative fluoroscopy image showing stabilisation of the long bone with a humeral CFR-PEEK nail.

This male patient in his 50s has been known to our orthopaedic department for years because of polyostotic fibrous dysplasia, making his bones susceptible to deformity and fracture [13]. He is a nonsmoker and takes regular pamidronate, tramadol, and naproxen. In the past, he had sustained stress fractures to his humeri, femora, and tibiae bilaterally, which were managed with conventional nails and plates (Figures 1(a), 1(d), and 1(e)). Despite an accurate surgical technique, achieving unity in fractures resulting from polyostotic fibrous dysplasia is difficult as normal bone has been replaced by fibroosseous tissue. Moreover, the analgesic benefit of non-steroidal anti-inflammatory drugs was at the expense of endochondral ossification. Indeed, over time, both his right tibial and left humeral plate-screw-osteosyntheses failed (Figures 1(a) and 1(e)). For both, the metalwork was removed and intramedullary stabilisation was performed. A 10 × 280 mm tibial and 8.5 × 240 mm humeral CFR-PEEK intramedullary nail with proximal and distal locking screws were used, respectively (Figures 1(b), 1(c), and 1(f)). The patient recovered well and was pain free in his right leg within one week post operation. His walking distance improved dramatically, and after 9 months, his nail has not failed.

## 3. Case Presentation II: Compatibility with Postoperative Radiotherapy

**Figure 2:**
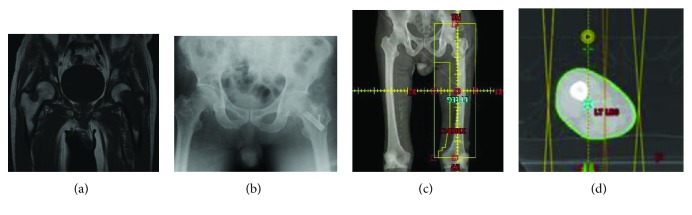
(a) Coronal T1 MR image showing extensive disease in the left femoral head, neck, and proximal metaphysis without an actual fracture of the bone. A contralateral lesion is seen in the superolateral aspect of the right femoral neck with several deposits along the femoral shaft. (b) AP pelvic X-ray showing prophylactic nailing with a CFR-PEEK cephalomedullary nail. (c, d) CT planning images for radiotherapy with the absence of metal artefacts.

This male patient in his 70s with a past medical history of hypertension, type II diabetes mellitus, and chronic kidney disease had been previously diagnosed with multiple myeloma, a neoplasm arising from clonal proliferation of plasma cells [14]. Besides chemotherapy, he had also been successfully treated with radiotherapy for symptomatic deposits in his lumbar spine and chest wall [15]. Unfortunately, he was readmitted after routine blood tests revealed significantly elevated adjusted calcium levels. Follow-up imaging showed a large left femoral lytic lesion with an impending fracture (Figure 2(a)). He therefore underwent prophylactic stabilisation using an 11 × 380 mm CFR-PEEK cephalomedullary nail to the left femur (Figure 2(b)). Radiotherapy planning was straightforward (Figures 2(c) and 2(d)) as there were no metal artefacts from the nail, and 5 times 4 Gy fractions were administered postoperatively to the left femoral lytic lesion. His postoperative recovery was complicated by delirium, cholecystitis, fast atrial fibrillation, urinary tract infection, and ongoing hypercalcaemia. The patient ultimately died from his disease burden.

## 4. Case Presentation III: Compatibility with Postoperative Enhanced Imaging

**Figure 3:**
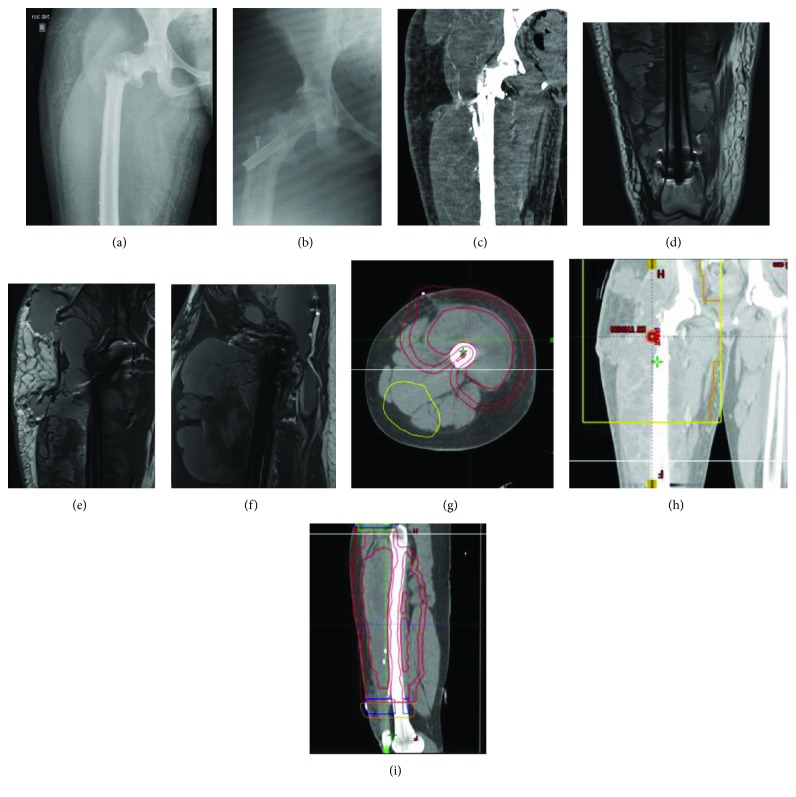
(a) Preoperative AP pelvic X-ray showing a pathological fracture through the trochanteric region of the right proximal femur. (b) Postoperative AP pelvic X-ray showing stabilisation of the fracture using a CFR-PEEK cephalomedullary nail. (c) Postoperative coronal CT image with minimal scattering showing massive progression of the right thigh sarcoma. (d–f) Postoperative MR images showing progression of tumour mass with an increase in cystic components but no signs of postoperative infection. (g–i) CT planning images for radiotherapy with the absence of metal artefacts.

This female patient in her 30s with no previous medical issues presented with a short history of pain and swelling in her right thigh without any preceding trauma. An MRI scan showed the presence of a haemorrhagic tumour over 20 cm in the craniocaudal dimension lying within the vastus intermedius muscle and encircling the right femur. The biopsy confirmed a myxoid spindle cell sarcoma with features consistent with myxofibrosarcoma, and CT of the chest, abdomen, and pelvis showed a nodule in the right lower lobe of the lung. She subsequently underwent excision of the tumour, unfortunately complicated by a pathological fracture of the trochanteric region of the right proximal femur noted after a fall (Figure 3(a)). It was stabilised using an 11 × 380 mm CFR-PEEK cephalomedullary nail (Figure 3(b)). The postoperative course was complicated by a superficial wound infection due to a Klebsiella pneumoniae. Given her immunocompromised status and pre-existing deformity of her leg, it was difficult to assess whether there was a deeper implant-related infection. Due to the lack of metal artefact from the CFR-PEEK nail, it was possible to perform an MRI scan and therefore deep infection could be ruled out (Figures 3(d)–3(f)). It did however show progression of tumour mass with an increase in cystic components. After wound healing, the patient was started on chemotherapy, and after the completion of two cycles, a CT chest, abdomen, and pelvis was repeated to assess response. Sadly, an increase in the size and number of the pulmonary metastases and a massive size progression of the known right thigh sarcoma were seen (Figure 3(c)). She then underwent palliative radiotherapy on her right thigh. Five fractions over seven days with a total dose of 20 Gy were given using anterior and posterior parallel opposed post fields (Figures 3(g)–3(i)) and provided benefit to both the pain and swelling she experienced. With no further surgical and medical options available, the focus became palliative care for symptom control. The patient subsequently died from complications resulting from her condition.

## 5. Discussion

For the majority of long bone fixations, titanium alloy and stainless steel nails will produce satisfactory outcomes. There are, however, specific clinical scenarios in which the biomechanical properties of CFR-PEEK nails can address the pitfalls associated with these types of nails. This would mainly involve patients receiving prophylactic and pathological fracture fixation for benign or metastatic disease who will undergo further advanced imaging or radiotherapy and/or in whom fracture healing might be delayed or absent. In this group of patients, the ability to reliably stabilise the limb, restore weight-bearing activity, and cause minimal disruption to adjuvant (oncological) management will have a positive impact on function and quality of life [16–18].

Case I demonstrates the use of CFR-PEEK nails in patients with non-malignant bone disease who have long life expectancy. As fracture healing is uncertain and bone quality might be poor, failure of conventional plates and nails or adjacent bone over time is not unlikely. Therefore, intramedullary stabilisation of the entire long bone using a nail with superior mechanical toughness is pragmatic. While biomechanical research has shown favourable properties of CFR-PEEK nails, long-term clinical data is still emerging. Of note, CFR-PEEK nails can fail as well as was recently shown in a case report by Loeb et al. [19]. They report mechanical failure of a CFR-PEEK nail at 10 weeks, which was used to treat a distal one-third tibia fracture in a patient with a history of smoking, vitamin D deficiency, and multiple previous low energy fractures. The authors provide some useful guidance on closed extraction of the implant in this unfortunate situation.

Case II demonstrates the use of CFR-PEEK nails in patients with metastatic disease who will receive radiotherapy after stabilisation of (impending) long bone fractures. The composite causes minimal artefacts on CT enabling better planning [12]. With less backscatter due to increased homogeneity of the field, lower doses are more effective. This reduces risks of non-union, wound complications, and potential adverse effects of radiotherapy on surrounding tissues [1].

Case III demonstrates the use of CFR-PEEK nails in patients with oncologic disease who will require postoperative enhanced imaging for assessment of response to adjuvant treatment. Reliable assessment of immediate periprosthetic tissues also allows for evaluation of possible deep infection in patients who are difficult to assess clinically. Although Metal Artefact Reduction Sequence (MARS) MRI can reduce artefacts of conventional nails to some extent, imaging results will still be inferior to those obtained after CFR-PEEK nailing. In vitro and clinical studies have quantified the artefact produced on MRI and CT following CFR-PEEK versus titanium nailing. Substantially less artefact was found in the CFR-PEEK nail group [9, 10].

CFR-PEEK is of course also radiolucent during intraoperative fluoroscopy, and therefore, a technical note is in place. A slight alteration of the insertion technique is required in comparison to, for example, titanium alloy nails. Attention must be paid to the tantalum markers for correct nail and screw placement. Arguably, placement of the cephalic screw for the cephalomedullary nail may indeed be easier, since the nail does not obstruct the fluoroscopic view of the trajectory of the guidewire.

Despite these advantages outlined above, the main limitation to the use of CFR-PEEK nails has traditionally been the prohibitive cost of the implant in comparison to conventional nails. However, with improvements in the manufacturing process combined with increasing usage and competitive cost structures provided by the industry, this is no longer the case [20, 21]. At our institution, the long cephalomedullary CFR-PEEK nail is approximately 35% more expensive than the equivalent titanium nail. Furthermore, the benefits of reduced artefact with advanced imaging and greater accuracy and safer dosing of radiotherapy arguably already outweigh these additional costs in appropriately selected patients.

Although we were unable to report on the long-term clinical outcomes for the cases presented, the clinical indications that are best suited to CFR-PEEK nailing tend not to produce long-term clinical information. This highlights the vulnerability of this group of patients and therefore the necessity of a nail that disturbs postoperative management as little as possible. Intermediate results at median 9 months of follow-up have reportedly been favourable [11]. With increasing availability and usage, future comparative studies should become possible. Randomized trials comparing, for example, fracture healing, amount of radiotherapy dosage used, and quality of life between groups of patients treated with CFR-PEEK and conventional nails would be of great interest to the orthopaedic surgeon.

## 6. Conclusion

CFR-PEEK nails have biomechanical properties potentially capable of overcoming disadvantages of conventional metal nails. Three case examples were presented which required superior mechanical toughness and compatibility with radiotherapy and postoperative advanced imaging. Although conventional metal nails will remain the gold standard for most long bone fixations, CFR-PEEK nails have a niche role in distinct groups of patients, specifically in prophylactic and pathological fracture fixation.
